# A new technique for mandibular osteotomy

**DOI:** 10.1186/1746-160X-3-15

**Published:** 2007-03-13

**Authors:** Edela Puricelli

**Affiliations:** 1 School of Dentistry, Federal University of Rio Grande do Sul, Porto Alegre, RS, Brazil

## Abstract

Sagittal split osteotomy (SSO) is a surgical technique largely employed for mandibular mobilizations in orthognatic procedures. However, the traditional design of buccal osteotomy, located at the junction of mandibular ramus and body, may prevent more extensive sliding between the bone segments, particularly on the advance, laterality and verticality of the mandibular body. The author proposes a new technical and conceptual solution, in which osteotomy is performed in a more distal region, next to the mental formamen. Technically, the area of contact between medullary-cancellous bone surfaces is increased, resulting in larger sliding rates among bone segments; it also facilitates the use of rigid fixation systems, with miniplates and monocortical screws. Conceptually, it interferes with the resistance arm of the mandible, seen as an interpotent lever of the third gender.

## Background

Osteotomies of the mandible have fundamental importance for correction of dental facial deformities (ICD K07). Osteotomy of the condylar neck was originally introduced by Jaboulay and Bérard in 1898 (apud Caldwell and Letterman, 1954) [1], and received important contributions by Babcock in 1909 [2].

Osteotomies of the mandibular ramus are currently preferred to osteotomies of the mandibular body. Their main advantages are related to lower risk of damage to the inferior alveolar neurovascular bundle, maintenance of extension of the mandibular body and no need for tooth extraction. They also allow for better aesthetic results in the region of the mandibular angle, through correction of the obtuse angle which characterizes prognathism [1].

Sagittal ramus osteotomy is one of the most efficient of these techniques [3]. The original designs for sagittal ramus osteotomy, performed with extra-oral access and involving a horizontal cut above the lingula, presented problems related to the small surface of contact between the resulting bone segments. Complications such as open bite and pseudarthrosis were usually a consequence of the procedures. Since the suggestion of cuts with inclined orientation by Kazanjian [4], the technique received a number of improvements. Schuchardt (apud Obwegeser) [5] suggested cutting the medial cortical surface of the ramus above the lingula, and the external surface 10 mm below the first cut. Trauner and Obwegeser [6] and Obwegeser [7] suggested that this distance should be increased to 25 mm, allowing for a larger area of contact. They were also responsible for the introduction of intra-oral access for performance of the technique.

Dal Pont [8] modified Obwegeser's method with the introduction of retromolar osteotomy. This alteration resulted in smaller displacement of the proximal segment due to muscle activity (jaw elevator muscles), so that the method could be used for other anomalies besides prognathism, such as retrognathism and open bite. Retromolar osteotomy was performed at the distal level of second molar, from the external oblique line to the inferior border of the mandible. The author proposed two types of fracture. For the sagittal type, the fracture extends to the posterior border of the ramus, and the masseter and medial pterygoid muscles are inserted in the proximal and distal fragments respectively. For oblique osteotomy, the path of medial fracture is within the mylohyoid groove, and both muscles are inserted into the proximal fragment.

Hunsuck [9] suggested that medial osteotomy should be extended up to the posterior region of the lingula, with no need for involvement of the posterior border of the ramus. Lateral osteotomy, on the other hand, according to his suggestion was performed at the junction of the ramus and body of the mandible.

Gallo, Moss and Gaul [10] introduced a modification to the Dal Pont method, aimed at treating retrognathism. According to their suggestion, vertical retromolar osteotomy of distal fragment starts near the external oblique line, extending through half the distance to the basilar region. The osteotomy tracing is turned horizontally according to the desired orientation for mandibular advancement, defining a step larger than the planned advance. Vertical osteotomy is then resumed, in a more anterior position. The area of contact between the fragments is increased, allowing metal osteosynthesis in the region of the mandibular body. Furthermore, rotation of the proximal fragment is prevented.

Epker [11] suggested an important change to the Obwegeser and Dal Pont method, minimizing complications such as excessive oedema, neurological complications related to the inferior alveolar bundle, hemorrhage and avascular necrosis of the segments. According to this proposition, no blind posterior dissection and periostal stripping of the masseteric-pterygoid sling is done. The author suggested gentle dissection of medial tissue from ramus just above the lingula (not extending to the posterior border of the ramus) for visual inspection of the inferior alveolar neurovascular bundle and elevation up to the antegoniac incisure, without posterior extension. Osteotomy starts above the lingula, extending inferolaterally up to the inferior border of the mandible, as recommended by Hunsuck [9]. The inferior cut, on the other hand, completely involves the basilar region, which makes sagittal split easier.

The use of different types of reciprocating saws was introduced in the decade of 1980. This technology resulted in a reduction in size of equipments and blades, allowing their use in sagittal osteotomies of the mandible [12]. Some of the items, such as the blade for basilar cutting developed by Wolford and Davis Jr [13], were specifically designed for particular stages of surgery.

The methods for fixation of these bone segments evolved from wire osteosynthesis. For rigid fixation in mandibular sagittal split ramus osteotomy, bicortical bone screws [14,15] and miniplates and screws [16-21] are now available.

## Surgical technique

The technique presented below has been in use since 1985. Performed under general anesthesia and nasotracheal intubation, the access is done through mucosal incision on the mandibular ramus, extending bilaterally below the mucogingival border beyond the mental foramen. Elevation is accomplished with conservation of the mental nerves. A channel retractor (Obwegeser type), positioned above the lingula, is used for medial access to the mandibular ramus. Two other channel retractors are employed for elevation of the buccal tissues of the mandibular body and ramus. One retractor for the ramus is placed on the temporal ridge, after partial elevation of this muscular insertion. Langenbeck retractors may be used for accessing the anterior region of the mandible. The areas are sequentially accessed, on both sides.

**Figure 1:**
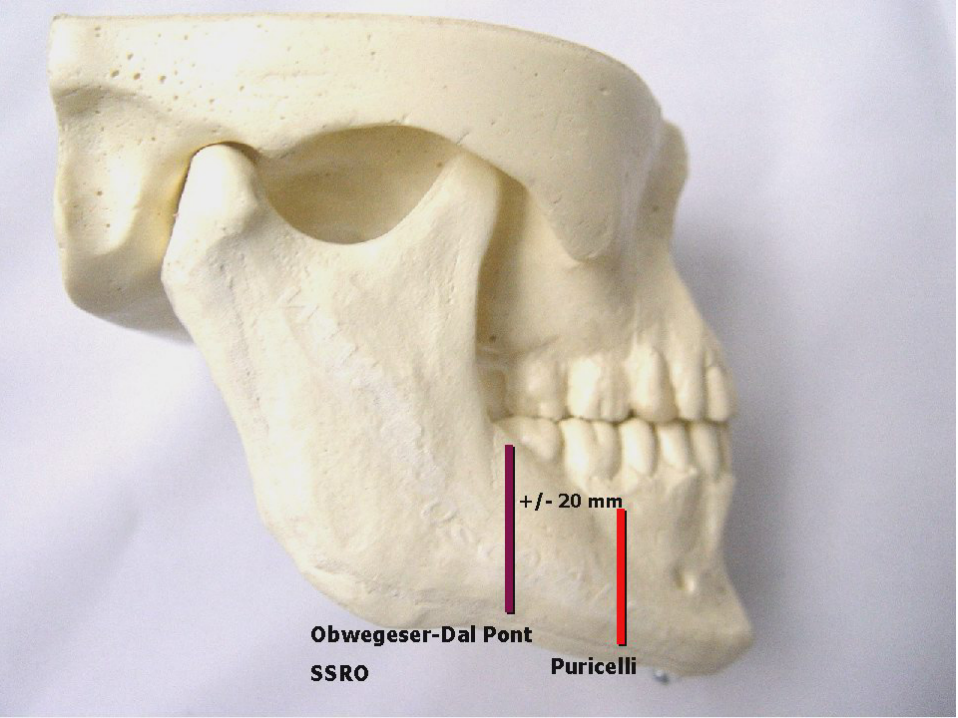
Laboratory model showing vertical osteotomies on the buccal side.

**Figure 2:**
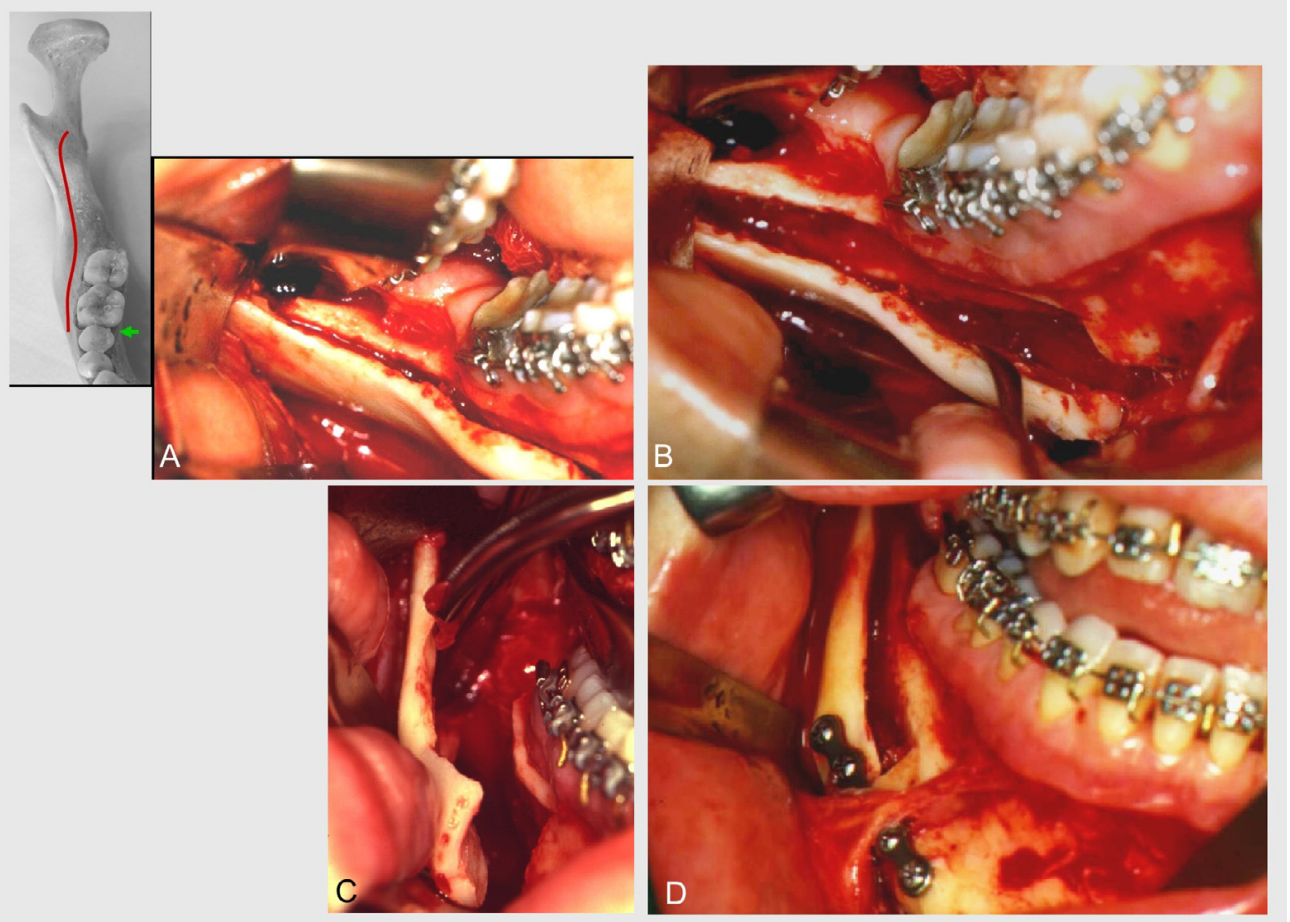
Transoperative characteristics. **A**. Osteotomy extending from medial aspect of the ascending ramus above the lingula, over the oblique external line to the mesial face of the permanent first molar. This cut is then extended vertically to the lower border of the mandible. The winding tracing is noteworthy. **B**. Separation of fragments after bone split. Larger extension of lateral segment and, in consequence, larger surface for bone contact. Exposition of the cruent area, including in its extension the neurovascular bundle; mental nerve. **C**. Complete liberation of osteotomized segments, allowing ample sliding between them. The lateral external segment of mandibular ramus and body is shown. Its extension and magnitude are noteworthy. Cruent area clearly visible in the depth of the bone surgical wound. Mandibular body. **D**. Application of miniplate and screw end surgical procedure in one of the sides. The sequence is repeated in the opposite side.

The technique presently suggested includes, as previous methods, medial osteotomy of the ramus, to be performed above the lingula and extending slightly behind it. An extension of sagittal osteotomy is performed on the buccal face, in anterior direction, making the lateral cut in the region of the mandibular body at the level of the mesial face of the first inferior molar. It is, therefore, up to 20 mm anteriorly located as compared to current protocols (Figure 1). Osteotomy is initially performed with small spherical and cylindrical burs, following the oblique external ridge up to the pre-determined level. The resulting osteotomy line will determine the orientation of the saw on the distal, more anterior direction. Distal continuity of the procedure involves a reciprocant saw, preventing damage to teeth roots (Figure 2A).

For splitting, osteotomes are sequentially malleted, beginning in the retromolar region with instruments oriented to the mandibular angle. Usual care procedures to avoid injury to the inferior alveolar bundle, particularly in the distal region, are of fundamental importance. Among them, maintaining lateral thickness of the proximal fragment, as well as orienting instruments in a direction parallel to the buccal cortical, are emphasized. Opening of the sagittal gap, from osteotomes placed on its most posterior region, allows the visual inspection of all its extension and loosening of the inferior alveolar bundle, in case it is exposed (Figure 2B, C).

After the fracture is completed, rigid internal fixation is performed with a 2,0 mm straight miniplate and monocortical screws. Its size will depend on the size of the planned movement (Figure 2D).

## Conclusion

Most studies and modifications proposed for mandibular sagittal split ramus osteotomy have concentrated in medial corticotomy. This is explained by the complexity of local anatomy and incidence of atypical fractures in this area, as well as by the frequent occurrence of neurological complications related to the inferior alveolar nerve. Following this stage of technical development, studies have concentrated in other limitations of the procedure, such as amplitude and direction of planned movements, particularly of laterolateral and vertical advance, alternatives for rigid fixation and stability of the results obtained. Other studies have also reported stability of the rigid fixation in sagittal osteotomy with the use of miniplates [19-21].

When we initiated the practice of fixation of mandibular surgeries with miniplates [18], the design usually employed in osteotomy was not adequate for their use. In experimental studies, the performance of mandibular bone cutting in a more anterior position was explored, in a process which resulted in the present proposal. The use of reciprocating saws facilitates the process, due to the curved shape of osteotomy and for prevention of damage to teeth roots

**Figure 3:**
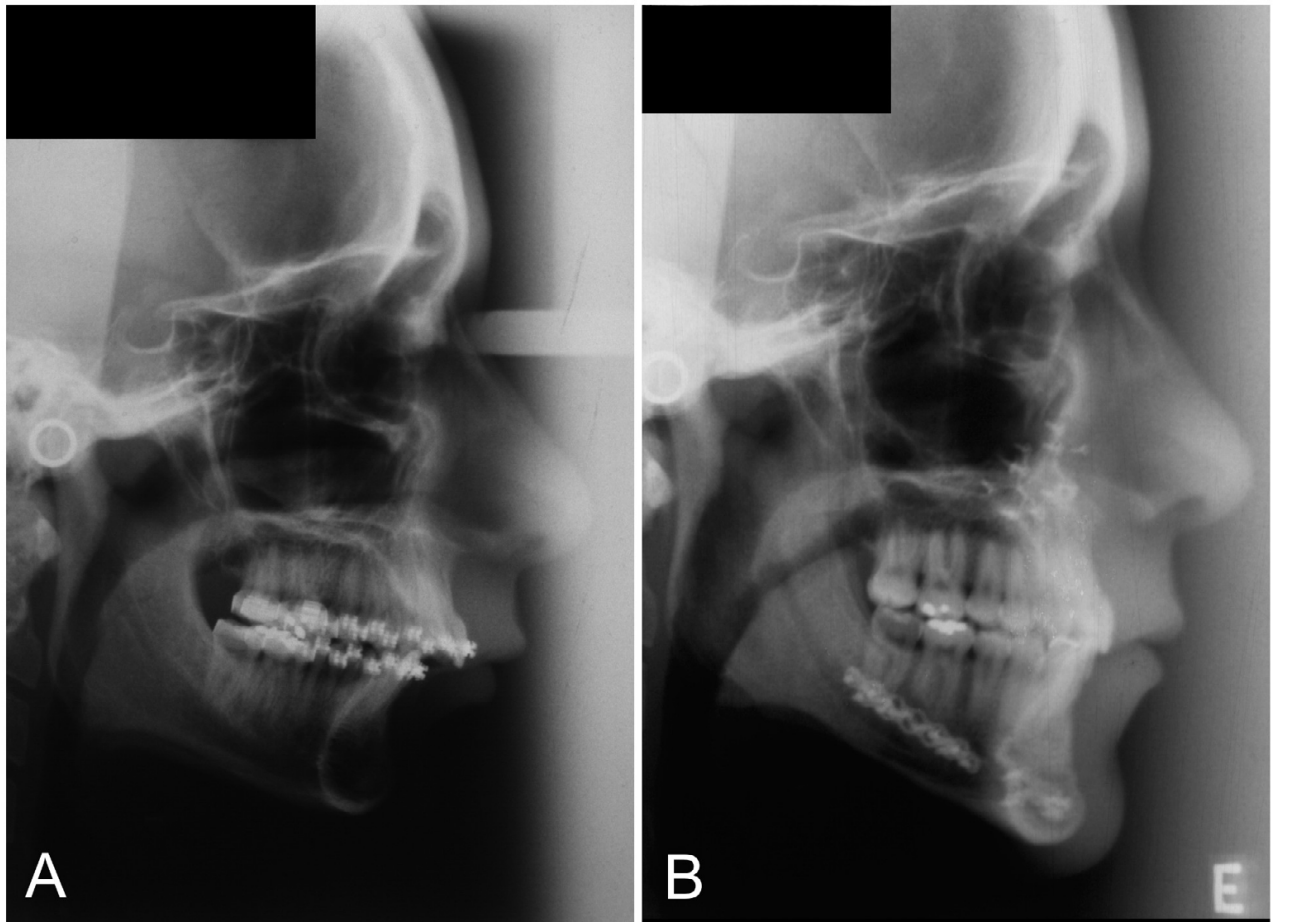
Radiographic comparison. **A**. Lateral extra-oral radiograph for preoperative cephalometric investigation of a retrognathic patient. Skeletal image of the retropositioned mandible can be seen in type II occlusal relationship. Soft structures show characteristic deep mentolabial sulcus and small facial height. **B**. Postoperative lateral extra-oral radiograph. Alveolar osteotomy can be seen from 32 to 42, associated to advancement and clockwise rotation of the mandible making up a maxilla-combined surgery. The surgery begins at the mandible. Rigid fixation miniplates measuring 2 mm are used in an extension of six holes for an advancement of 13 mm. On the maxilla, 1.5 mm rigid fixation may be observed. Soft tissue profile in accordance with skeletal results. In the naso-oro-hypopharyngeal regions, pre- and postoperative images show transversal increase of the area. This result is supported by respiratory improvement, as clinically reported by the patient.

**Figure 4:**
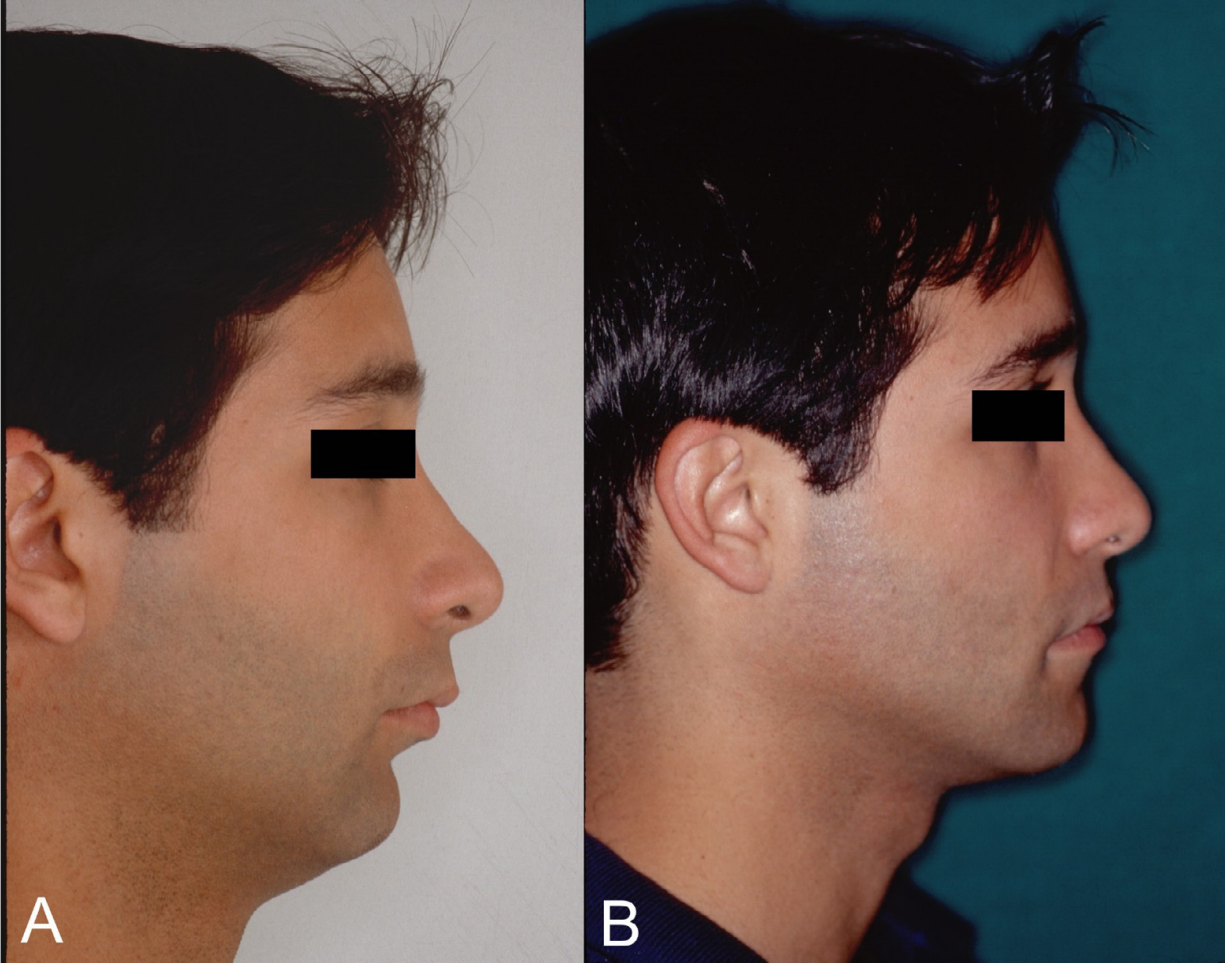
Lateral photographic study. E.A., male, 22 yeas old. Main complaint: poor dental occlusion and respiratory difficulty. **A**. Preoperative profile showing characteristic facial concavity of oral breather patient with class II occlusion. **B**. Postoperative profile: projection of the mentum, higher functional and skeletal balance in cervical angle and submental regions. Advancement and rotation of the maxilla results in projections at the malar and paranasal regions. Interlabial relationship improved. Elevation of the nasal point, resulting in more harmonious outline.

**Figure 5:**
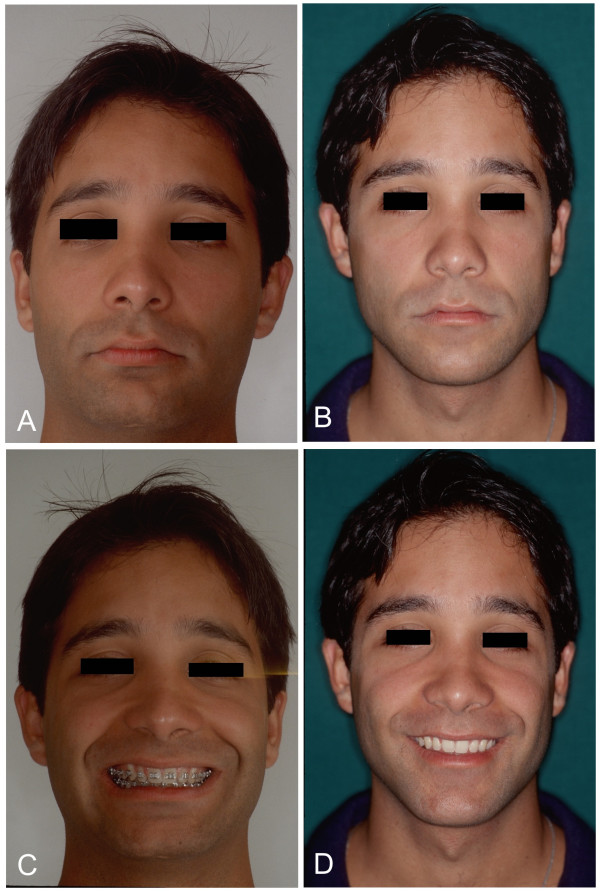
Frontal photographic study of the face. **A, B**. In the comparison of pre- and postoperative images, vertical increase of the mentum caused by advancement of the mandible with clockwise rotation is noteworthy. Lower incisive alveolar osteotomy, correcting dental projection, enhances correction of the mentolabial sulcus and allows greater advancement of the mandible. Better support of the median-third of face, with vertical and transversal symmetry patterns. **C, D**. Frontal view of smiling patient allows observation of functionality of the lips within aesthetic patterns. The oral corridor observed in the preoperative period was completely corrected.

**Figure 6:**
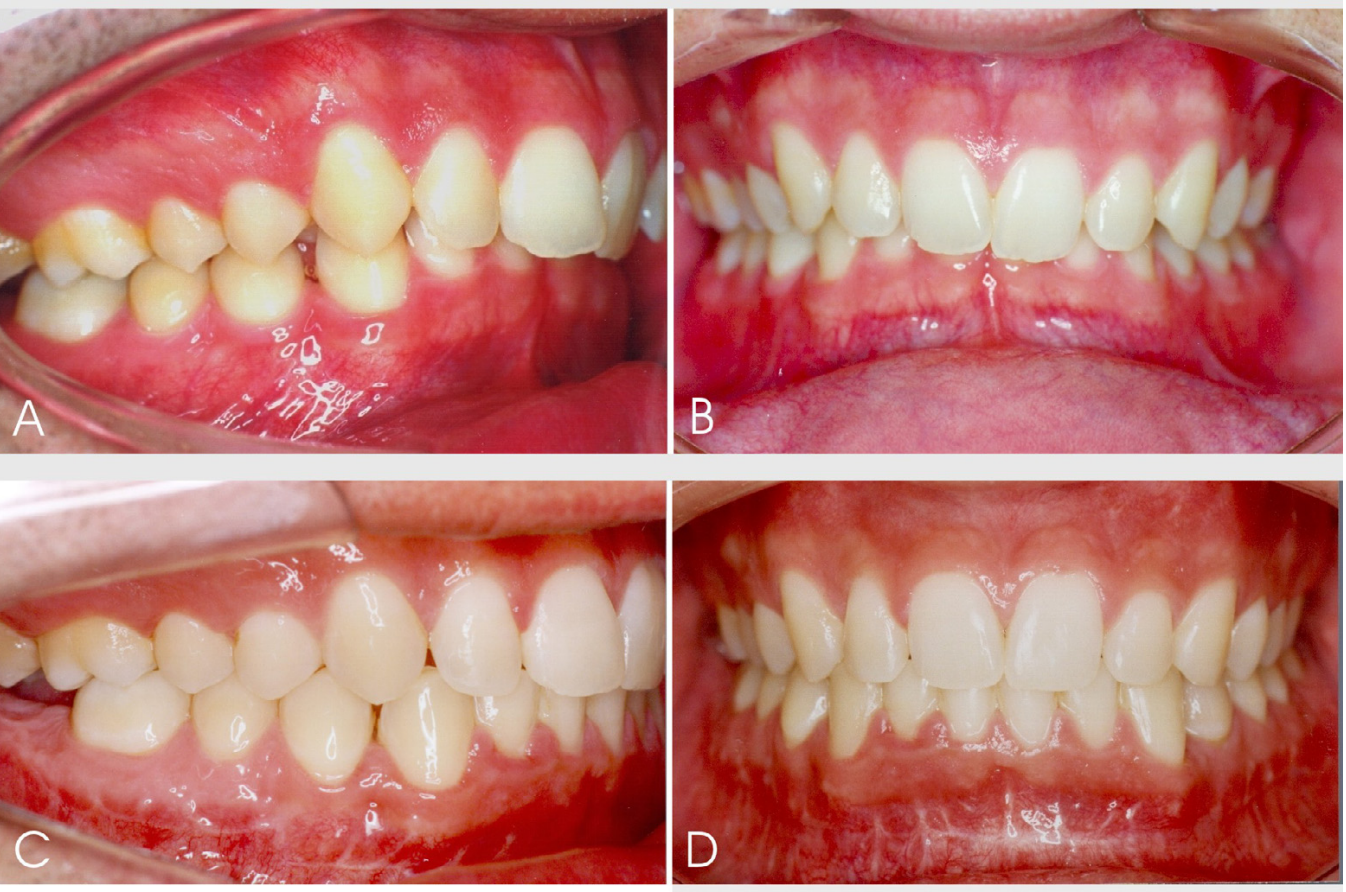
Dental occlusion. **A, B**. Preoperative: Patient reported for preoperative orthodontic treatment. Class II occlusion with deep bite, dental crowding and convergent inclination of posterior upper teeth are observed. Tooth 47 is lacking. **C, D**. Postoperative: dental occlusion surgical and orthodontic treatments completed with correction of deep bite. Dental alignment and levelling improve stability of surgical results, characterized by acquisition of molar and canine occlusion keys associated with good intercuspidation and interdigitation of the remaining dental structures.

This technical proposal presents many possible advantages. The area of bone contact is considerably increased, resulting in better healing, particularly in cases of great advance. Bone superposition is assured without interference with the area of fixation. The mechanical resistance decreases with anterior projection of osteotomy, lowering the burden of osteosynthesis. This is obtained through a 2,0 mm plate and monocortical screws (5 to 7 mm), placed in the region of the mandibular body. In this region, intraoral access is easier (avoiding the need of transcutaneous access for insertion of screws) and the flat bone surface facilitates adaptation of the plate. If there is interest in its removal, application of osteosynthesis in this area may also make it easier. In cases of simultaneous extraction of the inferior third molars, the fixation area is far from their alveoli and is not involved in the process. The same happens in atypical fractures that may eventually occur, involving the basilar region of the proximal fragment. The use of a larger miniplate will certainly give stability to the fragments (Figures 3 to 6).

Disadvantages of the technique involve the need for larger areas of elevation and manipulation of the mental nerve, since in many situations fixation of the plate will be performed in its proximity.

## Competing interests

The author(s) declare that they have no competing interests.
